# Supplementary material for: A Systematic Review and Meta-Analysis of Symptoms of Anxiety, Depression, and Insomnia in Spain in the COVID-19 Crisis
Source: Int J Environ Res Public Health. 2022 Jan 17;19(2):1018. doi: 10.3390/ijerph19021018 (PMC8775436; doi:10.3390/ijerph19021018)
Supplement: Supplementary file 1 [file ijerph-19-01018-s001.zip › ijerph-1378191-supplementary.pdf]

International Journal of
Environmental Research and Public Health

MDPI

Supplementary material

Table S1. Study characteristics for mental health symptoms in COVID-19 epidemic in Spain.

**Characteristics**	**Total Number of Studies/Samples ***	**Percent (%)**	**Level of Analysis**
Overall	28/38	100	
Design			Study
Cohort	4	14.29	
Cross-sectional	24	85.71	
Publication status			Study
Preprint	2	7.14	
Published	26	92.86	
Quality			Study
>6	7	25.0	
Between 5 and 6	21	75.0	
<5	0	0.0	
Population			Sample
Frontline HCW	3	7.89	
General population	30	78.95	
Student	5	13.16	
Outcome#			Prevalence
Anxiety	52	47.1	
Depression	52	47.1	
Insomnia	5	4.59	
Severity#			Prevalence
Above mild	39	35.78	
Above moderate	30	27.52	
Severe	23	21.1	
Overall	17	15.6	
	Median (mean)	Range	
Sample size	1199 (2272)	44 - 21207	Sample
Response rate	70.3% (73.9%)	20.0% - 98.0%	Sample
Female portion	70.25% (64.7%)	0% -100%	Sample

* One study may include multiple independent samples such as frontline HCWs and general population [31]. # The total prevalence of mental health outcomes are larger than the 38 samples because one sample can assess multiple mental health outcomes including anxiety, depression, and insomnia. Similarly, a study may report multiple levels of severity on each mental health outcome for each sample.

Table S2. The pooled prevalence rates of mental health symptoms by subgroups of population, outcome, and severity.

First-Level Subgroup	Second-Level Subgroup	Prevalence (%)	95% CI
	Aggregated prevalence	22%	18%–26%
Population	Frontline HCW	42%	22%–64%
	General population	19%	16%–23%
	Student	50%	32%–69%
Outcome#	Anxiety	20%	15%–25%
	Depression	22%	18%–28%
	Insomnia	57%	48%–66%
Severity#	Above mild	38%	30%–46%
	Above moderate	18%	14%–21%
	Severe	7%	5%–9%
	Overall	25%	16%–34%
Quality	Studies with high quality	21%	15%–27%
	Studies with medium quality	23%	19%–27%

Note: CI = Confidence Interval.
